# Acute exposure to caffeine improves foraging in an invasive ant

**DOI:** 10.1016/j.isci.2024.109935

**Published:** 2024-05-23

**Authors:** Henrique Galante, Massimo De Agrò, Alexandra Koch, Stefanie Kau, Tomer J. Czaczkes

**Affiliations:** 1 Animal Comparative Economics Laboratory, Department of Zoology and Evolutionary Biology, University of Regensburg, 93053 Regensburg, Germany; 2 Center for Mind/Brain Sciences (CIMeC), University of Trento, 38068 Rovereto, Italy; 3 Regensburg Center for Biochemistry (RCB), Laboratory for RNA Biology, University of Regensburg, 93053 Regensburg, Germany

**Keywords:** Biological sciences, Neuroscience, Behavioral neuroscience

## Abstract

Argentine ants, *Linepithema humile*, are a particularly concerning invasive species. Control efforts often fall short likely due to a lack of sustained bait consumption. Using neuroactives, such as caffeine, to improve ant learning and navigation could increase recruitment and consumption of toxic baits. Here, we exposed *L. humile* to a range of caffeine concentrations and a complex ecologically relevant task: an open landscape foraging experiment. Without caffeine, we found no effect of consecutive foraging visits on the time the ants take to reach a reward, suggesting a failure to learn the reward’s location. However, under low to intermediate caffeine concentrations ants were 38% faster with each consecutive visit, implying that caffeine boosts learning. Interestingly, such improvements were lost at high doses. In contrast, caffeine had no impact on the ants’ homing behavior. Adding moderate levels of caffeine to baits could improve ant’s ability to learn its location, improving bait efficacy.

## Highlights

• Intermediate doses of caffeine resulted in 38% shorter foodward foraging visits
• Improvement was driven by an increase in path straightness rather than speed
• Caffeine had no effect on ant homing behavior
• Adding caffeine to baits could increase recruitment and consumption of toxic baits

## Graphical abstract

**Figure:**
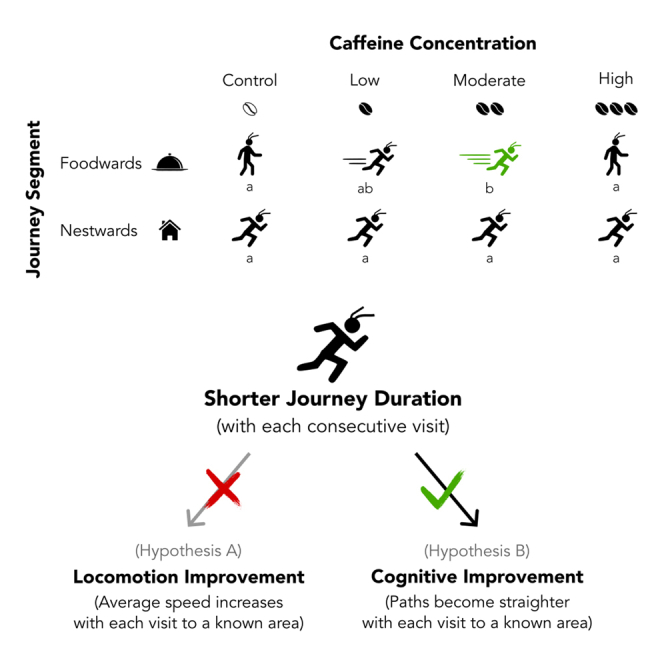

## Introduction

In Europe, the costs associated with invasive alien species are increasing 10-fold every decade, amounting to billions of euros.^1^ Among these invasive species, *Linepithema humile* (Mayr, 1868) is particularly damaging, considered one of the most ecologically harmful and costly invasive species worldwide^2^^,^^3^: invasive ants can outcompete native ants, leading to declines in biodiversity and abundance, which in turn, can have a variety of direct and indirect effects on other non-ant species.^4^^,^^5^^,^^6^ Given the severity of their impact, invasive ants are frequently considered a top priority for conservation and pest management programs.^7^^,^^8^^,^^9^^,^^10^

Studying foraging and navigation in *L. humile* can offer both fundamental insights into insect navigation, whilst potentially offering new approaches for their control. As central place foragers, ants must actively seek out food sources and bring them back to the nest. Navigation relies predominantly on two mechanisms: path integration and the use of learnt information. Path integration combines compass information^11^^,^^12^^,^^13^^,^^14^ with an odometer^15^ to continuously track the ant’s position relative to a reference point, usually either the nest or a frequently visited feeding site.^16^ This global vector allows ants to return back to the reference point even when navigating through featureless and novel environments. Simultaneously, many insects also employ view-based navigation. They are able to take a panoramic snapshot of a goal, such as their nest, a food source, or a point along a path and later compare it with their current view.^17^^,^^18^^,^^19^^,^^20^ Importantly, view-based navigation differs from path integration in that it does not exclusively rely on idiothetic cues. Instead, it is grounded in an individual’s ability to perceive and learn environmental cues.^21^ Nevertheless, both mechanisms greatly depend on an individual’s memory retrieval capabilities.^22^ However, whether these navigational mechanisms rely on the same neurological foundations,^23^^,^^24^ and the extent to which *L. humile* rely on these mechanisms remains unclear. Nonetheless, recent work highlights the use of path integration in other trail-laying species^25^^,^^26^^,^^27^ and *L. humile* has been shown to be a fast learner of multimodal cues.^28^^,^^29^^,^^30^

Pharmacological interventions offer an excellent tool for dissecting the neural and cognitive basis of behaviors such as navigation.^31^^,^^32^^,^^33^ Moreover, they provide the opportunity to manipulate, and potentially steer, the behavior of animals. This seems to have already been adopted by various plants, which spike their nectar with secondary metabolites, some of which influence neural activity.^34^^,^^35^^,^^36^ Such chemicals have the potential to artificially manipulate insect behavior.^37^^,^^38^^,^^39^ In this way, deploying neuroactives in artificial baits may be a promising way of enhancing control efficacy. If a bait additive could enhance the individual’s homing behavior, pioneer ants ingesting it might return to their nest faster, likely leading to increased recruitment to the bait relative to scouts which collected food without such additives. This in turn may result in baits with such additives outcompeting other food sources. Additionally, were the bait additive to improve the acquisition and use of learnt information during navigation, this could result in faster round trips to the bait, with each additional visit further reinforcing the pheromone trail, increasing recruitment to and consumption of the bait.^40^

Caffeine is especially interesting in this regard, as it is naturally occurring, cheap, and well-studied. In honeybees, it has been shown to elicit feeding preference^41^ and to enhance motivation and cognitive performance during complex learning tasks.^42^ Additionally, it increased foraging frequency and waggle dancing, quadrupling colony-level recruitment.^43^ Interestingly, it seems to hinder learning but not memory formation,^44^ although it is reported to improve long-term olfactory associations in honeybees.^45^ Similarly, caffeine improved bumblebee odor association learning^46^ and increased flower pollination.^47^ However, it lowered bumblebee food consumption.^48^ In ants, it likely alters food value perception, acting as both an attractant and a repellent, depending on the plant extract and concentration used.^49^^,^^50^^,^^51^ Furthermore, caffeine has been reported to improve learning and memory in *Myrmica sabuleti* ants, albeit at the cost of decreased food consumption.^52^

**Figure 1:**
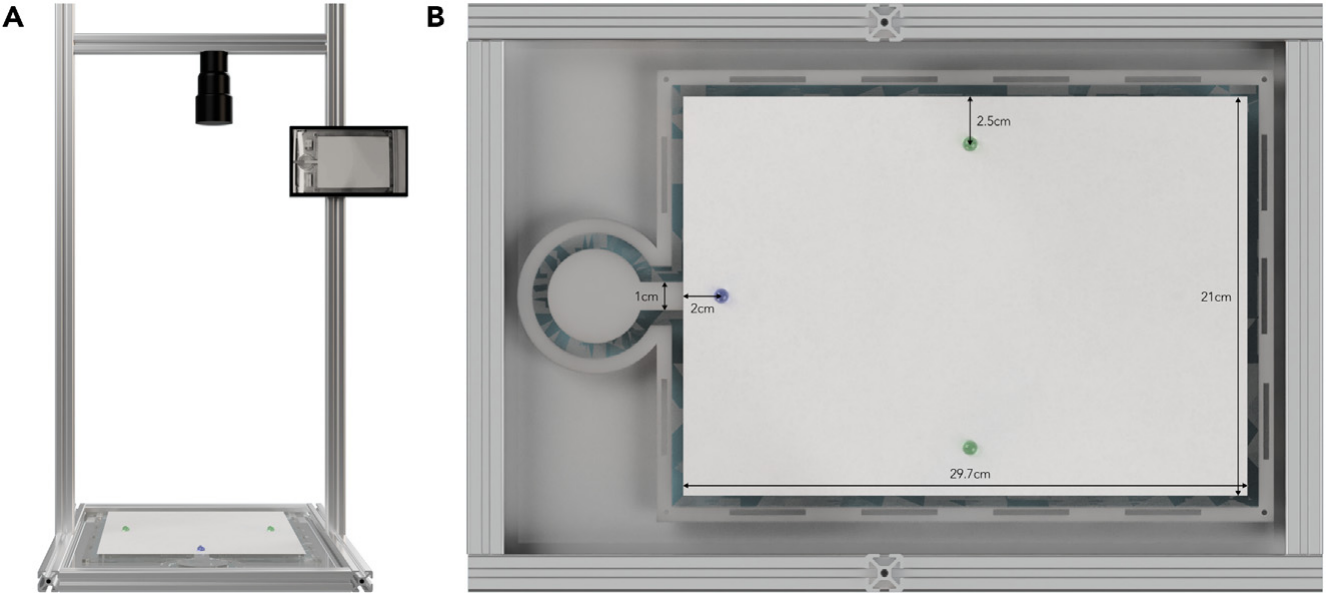
Open landscape experimental setup (A) Front view. A4 (210 × 297 mm) disposable paper overlay platform, surrounded by a water moat, with a single 1cm entrance, attached to a fixed top view Raspberry Pi HQ camera setup. (B) Top view. Blue drop represents the location of the sucrose solution drop (positive stimulus), either the control or the treatment on the ant’s first visit (2cm from the middle of the platform’s entrance). Green drops represent the possible location of the positive stimulus during the remaining visits (2.5cm from either the left or right side of the platform – each ant experienced the stimulus exclusively on one side).

Contrastingly, recent work suggests a general lack of effect of an intermediate concentration of caffeine on both spatial and olfactory associative learning in *L. humile*.^30^ However, that study focused on a single concentration of caffeine and overlooked its potential effects on foraging performance. Additionally, it tested learning in a binary choice Y-maze setup, which proved to be a very easy task for the ants, leading to a ceiling effect which further obscured the potential effects of caffeine. Here, we subject *L. humile* to a wider range of caffeine concentrations and a more complex and ecologically relevant task: an open landscape foraging experiment (Figure 1). Such a challenging task overcomes ceiling effect issues. By using an automated tracking system, we collected high resolution data of different segments of the foraging journey, as opposed to simple associative learning binary choices, studying not only the effect of caffeine on learning and memory, but also on locomotion and navigation.

## Results

There was no clear effect of caffeine concentration and/or the number of consecutive foraging visits to the landscape on the time ants spent drinking the reward (84s [min = 71s, max = 107s]).

### Low to intermediate caffeine concentrations lead to foodward path optimization

**Figure 2:**
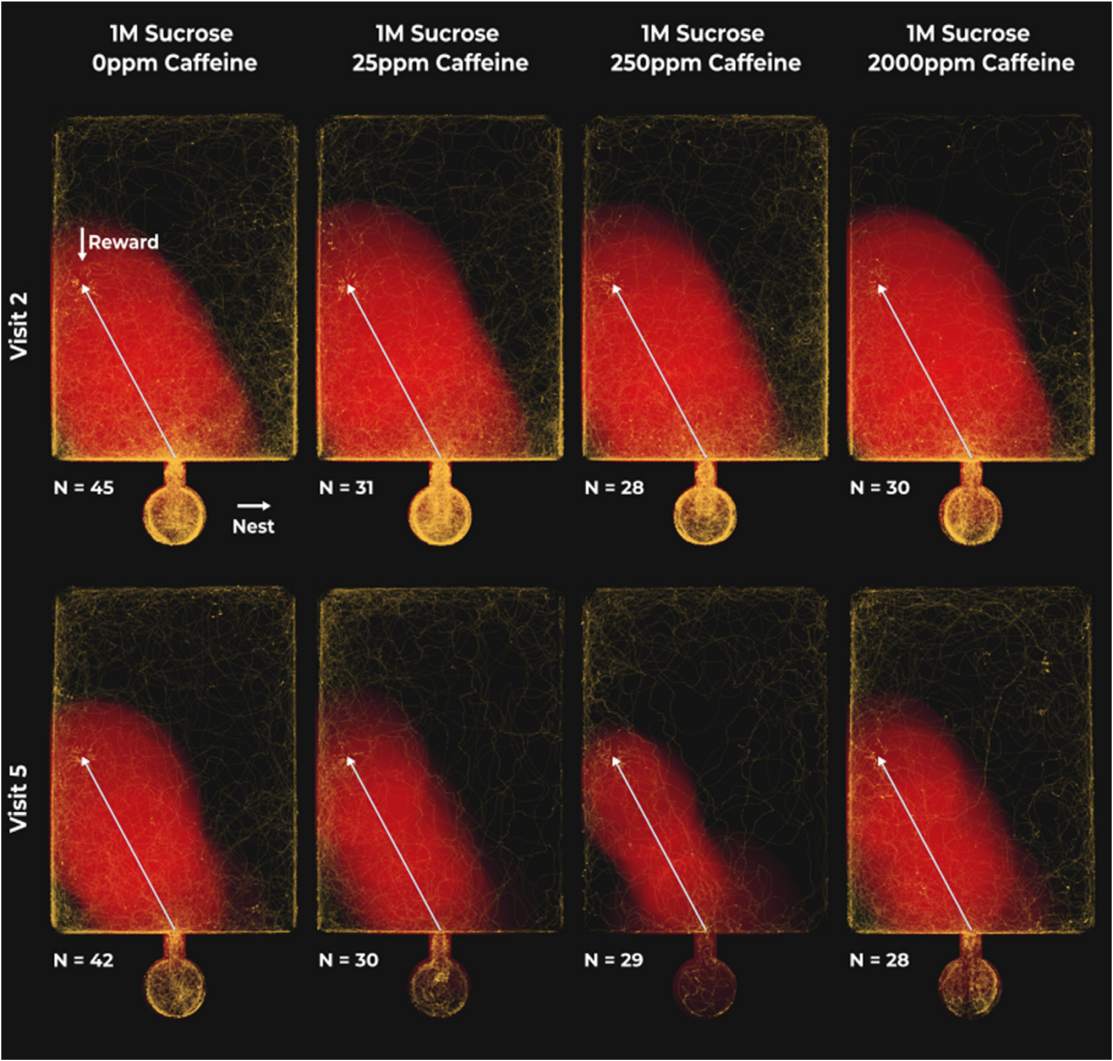
Dose-dependent effect of caffeine on consecutive foodward visits to the open landscape Red areas highlight the average deviation of all ant paths from the shortest route (white line) at each time point. Time normalization sets the starting point of each path at 0% and the final point at 100%, with 1% increments translating to 12s increments at most (less for shorter journeys). Larger areas surrounding the shortest path indicate larger deviations from it, while brighter regions suggest greater overlap between deviations at different time points implying a lack of directional movement. Note that closer adherence to the straightest line results in smaller, dimmer red areas, which translates into shorter times spent in the landscape and therefore fewer yellow points on the graph. Yellow points highlight the paths taken by individual ants, from first entering the landscape until first reaching the reward, with brighter spots indicating overlapping trajectories. To aid visualization, the paths of ants reaching the reward on the right side of the landscape were mirrored.

The time it took for an ant to reach the reward upon entering the landscape was influenced by both caffeine concentration and the number of consecutive visits to the landscape, as well as the interaction between the two (Figure 2; Table S1). On average ($\bar{x}$), across treatments and visits, the foodward journey took 212s [$\bar{x}$ min = 63s, $\bar{x}$ max = 372s].

**Figure 3:**
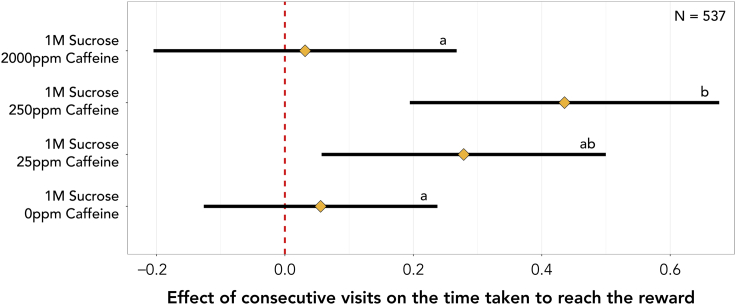
Effect of consecutive visits on the time an ant takes to reach the reward since first entering the landscape for each caffeine treatment Diamonds represent the estimated marginal means of linear trends obtained from the mixed effects cox proportional-hazards model and whiskers the respective 95% confidence intervals (see Table S2). Estimates of 0 (red dashed vertical line) indicate no effect of consecutive visits, whilst estimates >0 or <0 indicate that ants are more or less likely, respectively, to reach the reward faster with consecutive visits. If the 95% confidence intervals include an estimate of 0 it is likely that there is no effect of consecutive visits. Letters reflect statistical differences between treatments based on the estimated confidence intervals.

Under control conditions, ants showed little improvement in the time taken to reach the reward with every consecutive visit (5.6% [-12.6%, 23.8%, *N* = 537]). Meaning, if an ant initially took 300s to find the reward, after three consecutive visits to the open landscape, used henceforth as the example, it is likely to find the reward in 252s [133s, 428s]. However, when exposed to 25ppm of caffeine, each consecutive visit is likely to result in a 27.8% [5.7%, 50.0%, *N* = 537] faster foodward journey (113s [38 s, 252s]). This effect is almost doubled in the 250ppm caffeine treatment, where ants are likely to find the reward 43.5% [19.5%, 67.6%, *N* = 537] faster with every consecutive visit (54s [10s, 156s]). However, the benefits observed at lower to intermediate caffeine concentrations are lost when ants are fed 2000ppm of caffeine (3.1% [-20.5%, 26.8%, *N* = 537] | 273s [118s, 525s]). Furthermore, the effect of consecutive visits is 38.0% [7.8%, 68.2%, *N* = 537] higher in ants under the 250ppm caffeine treatment when compared to control-treated ants and 40.4% [6.6%, 74.2%, *N* = 537] higher when compared to the 2000ppm caffeine treatment (Figure 3; Table S2).

Notably, caffeine did not affect mean instantaneous speed during the foodward journey (Table S5). However, from the first (17.5 mm/s [min = 9.0 mm/s, max = 25.5 mm/s, *N* = 134]) to the last (18.6 mm/s [min = 7.4 mm/s, max = 27.7 mm/s, *N* = 129]) visit to the landscape, mean instantaneous speed increased by 0.3 mm/s [0.1 mm/s, 0.6 mm/s, *N* = 526] per visit (Table S6). The logarithm of path tortuosity remained constant with each consecutive visit both under control conditions (0.4 [0.0, 0.8, *N* = 526]) and under the 2000ppm caffeine treatment (0.4 [-0.1, 0.9, *N* = 526]). However, 25ppm (0.9 [0.4, 1.4, *N* = 526]) and 250ppm caffeine treatments (0.8 [0.3, 1.3, *N* = 526]) exhibited an increase in path straightness with each consecutive visit (Table S10). Importantly, effects in the logarithmic scale are often exponential in the linear scale. Meaning, if an ant had a path tortuosity of 20 on its first visit to the landscape, after three consecutive visits, we would expect it to have a path tortuosity of 6.0 [1.8, 20.0] under the control treatment, and a path tortuosity of 1.8 [1.0, 8.1] under the 250ppm caffeine treatment.

### Caffeine had no effect on the nestward journey

**Figure 4:**
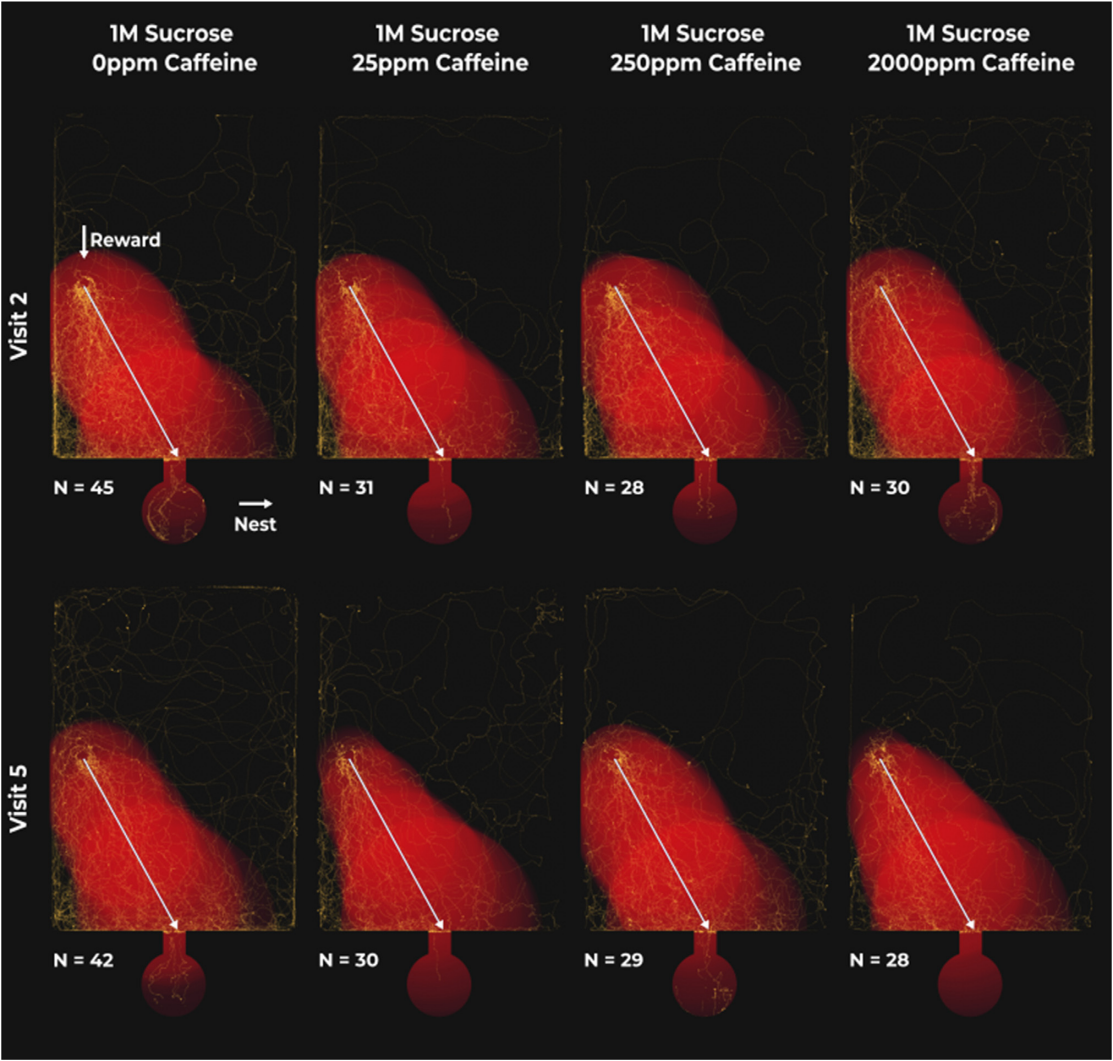
No effect of caffeine on consecutive nestward journeys Red areas highlight the average deviation of all ant paths from the shortest route (white line) at each time point. Time normalization sets the starting point of each path at 0% and the final point at 100%, with 1% increments translating to 2s increments at most (less for shorter journeys). Larger areas surrounding the shortest path indicate larger deviations from it, while brighter regions suggest greater overlap between deviations at different time points implying a lack of directional movement. Note that closer adherence to the straightest line results in smaller, dimmer red areas, which translates into shorter times spent in the landscape and therefore fewer yellow points on the graph. Yellow points highlight the paths taken by individual ants, from last touching the reward until leaving the landscape, with brighter spots indicating overlapping trajectories. To aid visualization, the paths of ants presented a reward on the right side of the landscape were mirrored.

The time it took for an ant to exit the landscape upon last touching the reward was influenced by the number of consecutive visits to the landscape, but not by the amount of ingested caffeine (Figure 4; Table S3). On average ($\bar{x}$), across treatments and visits, the nestward journey took 44s [$\bar{x}$ min = 30s, $\bar{x}$ max = 57s].

**Figure 5:**
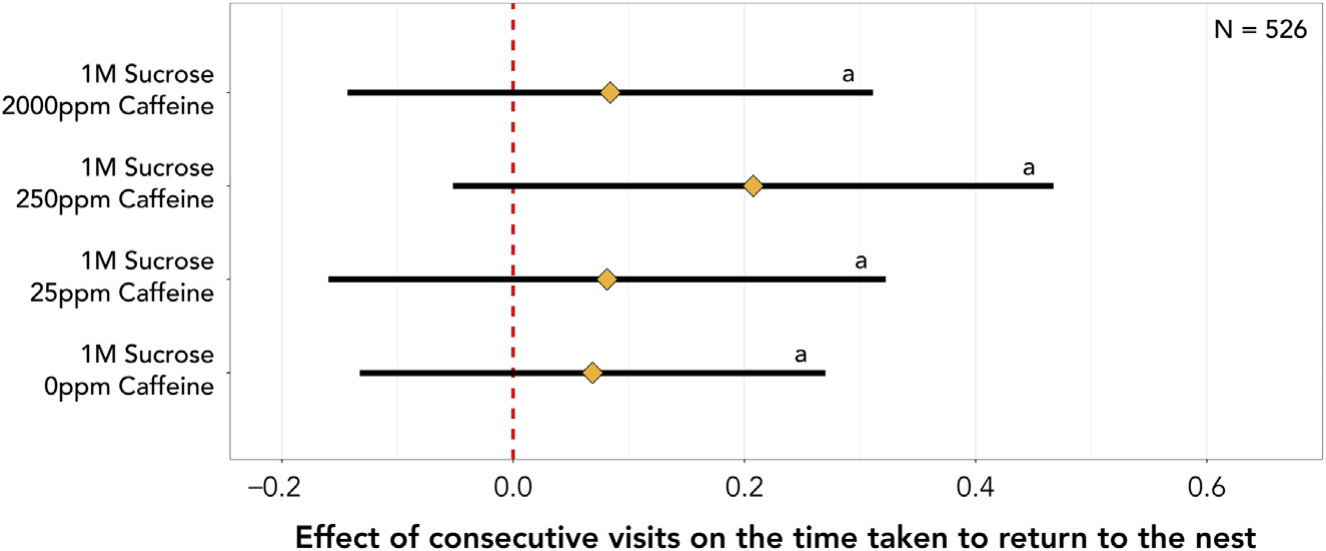
Effect of consecutive visits on the time an ant took to return to the nest since leaving the reward for the last time for each caffeine treatment Diamonds represent the estimated marginal means of linear trends obtained from the mixed effects cox proportional-hazards model and whiskers the respective 95% confidence intervals. Estimates of 0 (red dashed vertical line) indicate no effect of consecutive visits, whilst estimates >0 or <0 indicate that ants are more or less likely, respectively, to return to the nest faster with consecutive visits. If the 95% confidence intervals include an estimate of 0 it is likely that there is no effect of consecutive visits. Letters reflect statistical differences between treatments based on the estimated confidence intervals.

With each consecutive visit ants are likely to be 11.0% [2.8%, 19.2%, *N* = 526] faster at returning to the nest (Table S4). Meaning, if an ant initially took 30s to return to its nest, after three consecutive visits to the open landscape, it is likely to return in 21s [16s, 28s], regardless of its consumption of caffeine (Figure 5). Contrastingly, mean instantaneous speed (14.5 mm/s [min = 4.9 mm/s, max = 26.9 mm/s, *N* = 526]) was constant throughout consecutive visits and treatments (Table S7). Furthermore, albeit unaffected by caffeine, the logarithm of path tortuosity decreased by 0.07 [0.02, 0.11, *N* = 526] per visit (Tables S11 and S12). Therefore, if an ant had a path tortuosity of 4 on its first visit to the landscape, after three consecutive visits, we would expect it to have a path tortuosity of 3.2 [2.9, 3.8].

## Discussion

Low (25ppm) and intermediate (250ppm) concentrations of caffeine shortened the foodward journey over consecutive visits (Figures 2 and 3). This was not due to an increase of mean instantaneous speed, but rather an increase in path straightness with each consecutive visit under these treatments. Thus, caffeine is likely dose-dependently boosting learning, as a straighter path suggests the ant knows the location of the reward (Figure 2). However, the improvements gained were lost at high doses of caffeine (2000ppm). This suggests a hormetic dose-response pattern, where caffeine is toxic at high doses, but when ingested in smaller amounts has the opposite effect, stimulating biological function. Similar results were previously found in bees for a variety of chemicals,^53^ including caffeine.^45^ In honeybees, higher doses of caffeine have resulted in a reduced likelihood of bees to respond to a conditioned odor^44^ and 2000ppm of caffeine has previously been found as the LD_50_ of honeybees.^54^ In humans, low to moderate caffeine dosages often have stimulant and performance-enhancing effects. On the other hand, high doses may be associated with aversive somatic effects, including sleep disruption and increased anxiety and agitation, all of which can contribute to impaired fine motor control.^55^^,^^56^^,^^57^^,^^58^ In this way, it seems reasonable that the loss of effect at high doses of caffeine is likely due to its toxicity.

Interestingly, caffeine had no clear impact on nestward journey duration (Figures 4 and 5). Much as in the foodward journey, consecutive visits did not alter the speed at which the ants moved. However, ants moved slower, on average, during the nestward journey when compared to the foodward journey, possibly due to being heavier when their crop is full. Independently of caffeine, over consecutive visits nestward paths became straighter, which translated into shorter nestward journey durations across treatments. In part, the lack of effect of caffeine observed could be due to the nestward journey durations being an order of magnitude shorter than foodward journey durations. The asymmetry between the two journey segments could be a result of the ants often returning to the entrance of the open landscape during their foodward journey but taking fewer detours during their nestward journey. Thus, any positive effects caused by caffeine would be harder to detect as there is less room for improvement.

Nevertheless, in invaded areas, ants need to navigate more complex environments, relying on multimodal cues. Under such conditions, where learning and memory play a key role, caffeine might have an impact on the nestward journey as well. In fact, we note a slight trend toward faster nestward journeys over consecutive visits in the 250ppm treatment (Figure 5). The range over which panoramic views offer navigational guidance is smaller in denser environments and larger in open landscapes.^59^ In this way, under the experimental conditions used, ants are likely to have heavily relied on view-based navigation during the foodward journey. However, in their invasive range, and considering the temporary nature of their nests, *L. humile* habitats are likely to be denser and richer in landmarks. Therefore, ants will not only rely on visual cues but also olfactory ones. Caffeine has been shown to improve olfactory associations in both honeybees and bumblebees.^45^^,^^46^ Furthermore, ants have been shown to use olfactory landmarks to locate food and their nest^60^^,^^61^^,^^62^ with a combination of visual and olfactory navigational tools leading to increased performance.^63^ Thus, the observed navigational improvements are likely to not only be maintained, but potentially increased under natural conditions.

Physiologically, the lack of effect of caffeine during the nestward journey, in contrast to the foodward journey, implies caffeine selectively targets distinct navigational mechanisms. Path integration and view-based navigation are thought to be encoded by different brain regions.^64^ The central complex is thought to directly modulate locomotion and be responsible for path integration.^23^^,^^65^ On the other hand, view-based navigation and learning are thought to be predominantly based on the mushroom bodies.^32^^,^^33^ Nevertheless, new models suggest a more complex interaction of both with other brain regions during navigation.^66^ Considering the lack of effect of caffeine on locomotion during the entire foraging journey, paired with an increase in path straightness during the foodward journey but not the nestward journey, it is likely that caffeine predominantly affected the mushroom bodies, and therefore view-based navigation, over the central complex.

In fact, caffeine is known to interact with invertebrate ryanodine receptors, prompting the release of intracellular calcium stores.^67^ Pisokas et al.^68^ proposes a separation of the directional and learning components of path integration, suggesting that the foundation of path integration memory may involve molecular processes linked to CaMKII signaling pathways. These proteins are central to synaptic plasticity, learning and memory and an increase in calcium influx can rapidly enhance their activity. Moreover, studies have associated both CaMKII and caffeine with long-term memory formation in crickets.^69^^,^^70^ Combined with our results, these findings suggest caffeine is likely interfering with ant learning and memory.

Alternatively, low to intermediate caffeine concentrations could be acting by enhancing alertness and motivation. However, caffeine did not impact crop load or consumption rate in *L. humile*, suggesting it was either not perceived by the individuals or did not influence reward value (Galante et al. In prep.). Nevertheless, across treatments, ants have a strong tendency to stay close to the edges of the open landscape (positive thigmotaxis). This behavior appears to decrease under intermediate doses of caffeine (Figure 2, Visit 5) which could suggest caffeine is increasing foraging motivation. This could be due to the effects of caffeine as an adenosine receptor antagonist, potentially leading to increased levels of acetylcholine which might improve cognitive alertness.^71^ Moreover, a decrease of GABA, a neurotransmitter thought to impair olfactory memory in bees,^72^ might reduce forgetting of the location of the caffeinated rewards. In turn, this could generate addiction-like symptoms potentially increasing the ant’s motivation to forage on such rewards. However, a recent study exploring visual learning in bumblebees shows GABA promoting flower fidelity.^73^ Caffeine has been shown to increase responsiveness and activity in jumping spiders^74^ as well as having an effect on locomotion in a variety of insects.^67^ However, we show no effect of caffeine on ant mean instantaneous speed throughout the foraging journey, further suggesting caffeine-mediated foraging improvements are not a consequence of motor efficiency but rather of cognitive enhancement.

Similarly to Si, Zhang, and Maleszka,^42^ we found that caffeine has no effect in a simple Y-maze spatial and olfactory association learning task,^30^ but its effects become visible in a more complex and field-realistic experiment. Consecutive visits did not alter foodward journey duration in control treated ants (Figure 3). This implies that three visits to the reward were not sufficient for the ants to learn and/or memorize its location. However, previous work has demonstrated that *L. humile* are excellent spatial and olfactory learners often requiring as little as one visit to the reward to learn its characteristics and location.^28^^,^^29^^,^^30^ We were thus successful in creating a more complex and challenging experiment, which arguably better depicts natural conditions. Furthermore, this enabled us to exclude ceiling effects potentially observed in previous experiments which might have masked any effects resulting from exposure to caffeine.^30^ Ceiling effects are a major challenge of simpler learning setups, and open landscape tasks a suitable solution to this problem.

Intermediate concentrations of caffeine improved foraging in Argentine ants, demonstrating that adding neuroactive substances to toxic baits may hold potential as a novel approach to improve invasive insect control. Future work, ideally under natural conditions, should test whether caffeine interacts with the chosen toxicant, and if it also improves recruitment and visitation rates in ants*,* as previously shown in honeybees and bumblebees, respectively.^43^^,^^47^ As new invasive ant species start to become established worldwide,^75^ there is growing urgency in finding ways to improve control efforts. Adding low dosages of caffeine to toxic baits may be a cheap and easily deployable method of boosting learning of bait location, potentially leading to increased recruitment to and consumption of the toxicant, and ultimately improved control.

### Limitations of the study

In this study, we demonstrate that acute exposure to caffeine can lead to shorter times to reach a food source over consecutive foraging visits. Notably, such improvements are driven by straighter paths rather than increases in mean instantaneous speed, suggesting caffeine acts on learning processes. Nonetheless, our experimental setup does not allow us to definitively link the observed effects of caffeine with specific navigational mechanisms such as path integration or view-based navigation.

Moreover, the foraging distances traveled by the ants in our setup are orders of magnitude shorter than those they would travel in a natural environment. Thus, it is unclear if at longer foraging distances, where caffeine would remain in the ant’s system for longer, there would be a difference in its effects. Additionally, further work is required in order to understand if the effects reported here would be diminished or enhanced at a collective level where ants share food across the colony and make collective foraging decisions.

## STAR★Methods

### Key resources table

**Table:** 

REAGENT or RESOURCE	SOURCE	IDENTIFIER
**Chemicals, peptides, and recombinant proteins**		

Caffeine	Sigma-Aldrich (Taufkirchen, Germany)	CAS 58-08-2

**Deposited data**		

Data, preregistration and statistical analysis	This paper	https://doi.org/10.5281/zenodo.8413979

**Software and algorithms**		

DeepLabCut V2.2.2	https://github.com/DeepLabCut/DeepLabCut	https://doi.org/10.1038/s41593-018-0209-y and https://doi.org/10.1038/s41596-019-0176-0

### Resource availability

#### Lead contact

Further information and requests for resources and reagents should be directed to and will be fulfilled by the lead contact, Henrique Galante (gosocialants@gmail.com).

#### Materials availability

This study did not generate new unique reagents.

#### Data and code availability

• All original data have been deposited at Zenodo and are publicly available as of the date of publication. DOI is listed in the key resources table.
• All original code has been deposited at Zenodo and are publicly available as of the date of publication. DOI is listed in the key resources table.
• Any additional information required to reanalyse the data reported in this paper is available from the lead contact upon request.

### Experimental model and study participant details

#### Colony maintenance

*Linepithema humile* (Mayr, 1868) were collected from Portugal (Proença-a-Nova) and Spain (Girona) between April 2021 and January 2022. Ants were split into colony fragments (henceforth colonies), containing three or more queens and 200–1000 workers, kept in non-airtight plastic boxes (32.5 × 22.2 × 11.4 cm) with a plaster of Paris floor and PTFE coated walls. 15mL red transparent plastic tubes, partly filled with water, plugged with cotton, were provided as nests. Ants were maintained on a 12:12 light:dark cycle at room temperature (21°C–26°C) with *ad libitum* access to water. Between experiments, ants were fed *ad libitum* 0.5M sucrose solution and *Drosophila melanogaster* twice a week. During experiments, ants were fed once a week and deprived of carbohydrates for four to five days prior to testing, ensuring high foraging motivation. Experiments were conducted between September 2021 and March 2022 using 14 colonies divided into donor/recipient pairs. Focal ants left their original colony (donor) but returned to a different colony (recipient) to unload the contents of their crop. This ensured donor colonies, and consequently focal ants, were never exposed to caffeine prior to the experiment. We have conducted all experiments in accordance with the guidelines that are applicable to working with the model organism in the European Union. Colonies were kept in closed boxes under oil baths in order to prevent any escape.

### Method details

#### Chemicals and solutions

Caffeine (CAS 58-08-2) was obtained from Sigma-Aldrich (Taufkirchen, Germany). 1M sucrose solutions (Südzucker AG, Mannheim, Germany) mixed with different caffeine concentrations were used as treatments. Identical 1M sucrose solutions were used as controls. Caffeine concentrations were chosen based on previous reports of their effects on Hymenopterans. Caffeine solutions ranged from a low, naturally occurring concentration^41^^,^^43^ of 25ppm (0.13 μmol mL^−1^) to an intermediate concentration of 250ppm (1.29 μmol mL^−1^), and a high concentration of 2000ppm (10.30 μmol mL^−1^), previously reported as the LD_50_ of honeybees.^54^ A double-blind procedure was applied to all solutions used in order to minimize experimenter bias.

#### Open landscape spatial learning

A challenging and ecologically relevant experimental paradigm was developed to study the effects of different concentrations of caffeine on spatial learning and memory. This setup consisted of an A4 (210 × 297 mm) disposable paper overlay platform, surrounded by a water moat, with a single 1cm entrance, attached to a fixed top-view Raspberry Pi HQ camera setup. An *ad libitum* sucrose solution drop (positive stimulus), either pure or laced with 25ppm, 250ppm or 2000ppm of caffeine, was positioned at the entrance of the platform (see blue drop in Figure 1B). A single ant from a donor colony, placed on the table to the right of the setup, was allowed onto the platform via a mobile drawbridge, was marked with acrylic paint while drinking, and returned to its paired recipient colony. Meanwhile, the disposable overlay was replaced, ensuring the removal of any pheromone trails laid, and a new *ad libitum* drop of the solution used during the first visit placed on one of the sides of the platform (see green drops in Figure 1B). After unloading, the ant was placed back onto the platform’s entrance where it was video recorded and the time it spent in the colony, the time it took to reach the reward, to drink and to return to the colony was noted for each visit. Ants were not prevented from using available room cues, as for example the ceiling light. Overall, each ant carried out five consecutive visits to the platform: a priming visit, with the reward placed at the entrance of the platform, followed by four visits with the reward either always on the left or the right side of the landscape. 142 individuals were tested, with a complete run taking between 35 and 155 min.

### Quantification and statistical analysis

#### Data extraction and processing

DeepLabCut version 2.2.2^76^^,^^77^ was used to automate video analysis, allowing for the acquisition of coordinates for each ant’s head and gaster, as well as those of each corner of the A4 platform and the centre of the solution drop at every frame (videos recorded at 30fps). In total 142 ants were recorded, however five of them were excluded as they were unable to find the reward within 20 min in at least two of their five visits to the landscape. Furthermore, 11 ants failed to locate the reward in one of their five visits and one other was lost in the colony before it could do its final visit, therefore those visits were removed. 10 other visits were excluded due to tracking associated issues (specified in the detailed statistical analysis and code). Overall, 526 visits of 136 ants were analysed. Python version 3.7.13^78^ was used to standardise the ants’ coordinates by ensuring the same corner of the A4 platform was used as the origin of the cartesian referential of all videos. The known dimensions of the A4 were further used to convert coordinates from pixels to millimetres. To account for DeepLabCut tracking errors, any ant movement exceeding two millimetres per frame was considered implausible and subsequently removed. This is due to biological constraints that limit ants' movement to a maximum speed of around one millimetre per frame.^79^ In this process, an average of 0.1% [0%, 0.8%, *N* = 526] of frames were eliminated from each video. The times at which an ant reached and left the reward were automatically derived from the tracking data. These were used to define the ant’s foodward and nestward journeys. The foodward journey encompassed the time from when the ant initially entered the landscape until it reached the reward for the first time. The nestward journey was taken as the last time the ant left the reward until it left the landscape. For each visit, the mean instantaneous speed and path tortuosity were then calculated separately for both the foodward and nestward journeys. Mean instantaneous speed represents the average distance travelled by the ant within a frame (1/30s), while path tortuosity quantifies the total distance covered by the ant relative to the minimum possible distance it could have travelled, providing a measure of path straightness. Importantly, exclusion criteria and extracted variables were defined *a priori* in a pre-registration with a few notable deviations. Mainly, the 25ppm and 250ppm caffeine treatments were added at a later stage and the second visit to the landscape was analysed together with all others in order to better understand the effect of consecutive visits on navigation.

#### Statistical analysis

The complete statistical analysis output, and the entire dataset on which this analysis is based, is available from Zenodo (https://doi.org/10.5281/zenodo.8413979).

All graphics and statistical analysis were generated using R version 4.2.1.^80^^,^^81^^,^^82^^,^^83^ Foodward and nestward journey duration were analysed using mixed effects cox proportional-hazards models.^84^^,^^85^ Note that for the survival analysis of foodward journey duration the 11 ants which failed to find the reward in one of their five visits were included yet censored. Mean instantaneous speed and path tortuosity were analysed with linear mixed-effects models.^86^ DHARMa^87^ was used to assess linear model assumptions and MuMIn^88^ to obtain a measure of goodness of fit. Analysis of variance tables were used to test the effects of the regressions coefficients.^89^ Estimated marginal means of linear trends and contrasts were obtained using the emmeans package^90^ with Bonferroni adjusted values accounting for multiple testing. Consecutive visit effects were assumed to be relatively linear as previously shown in Y-maze experiments performed on *L. humile*.^29^^,^^30^ We avoid the use of p-values, and their associated binary decision of significant/nonsignificant, instead reporting effect size estimates and their respective 95% confidence intervals shown throughout the results section as (estimate [lower limit, upper limit, N = sample size]). Nevertheless, should they be of interest, these are reported in the supplementary materials.
