# Supplementary material for: Acute exposure to caffeine improves foraging in an invasive ant
Source: iScience. 2024 May 23;27(6):109935. doi: 10.1016/j.isci.2024.109935 (PMC11270030; doi:10.1016/j.isci.2024.109935)
Supplement: Document S1. Data S1 and Tables S1–S12 [file mmc1.pdf]

iScience, Volume *27*

Supplemental information

Acute exposure to caffeine improves foraging in an invasive ant

Henrique Galante, Massimo De Agrò, Alexandra Koch, Stefanie Kau, and Tomer J. Czaczkes

Data S1 - The following data, preregistration, and statistical analysis (related to STAR Methods) are publicly available from Zenodo (https://doi.org/10.5281/zenodo.8413979).

- **sample_videos.zip**: A subset of the videos used for data extraction. The complete collection of videos is not publicly accessible primarily due to their considerable size (105.35GB). Requests for access to the entire video set are encouraged.
- **Preregistration.pdf**: The preregistration created for data collection and analysis with justifications for deviations from it.
- **OpLan_D1_metadata.csv**: Manually collected metadata pertaining to experimental conditions, subjects, and treatments.
- **OpLan_D2_DLC_coordinates.zip**: Cartesian coordinates obtained from DeepLabCut for each of the videos analysed.
- **OpLan_C1_reproject_coordinates.py**: Python code used to standardise the ants' coordinates by ensuring the same corner of the A4 platform was used as the origin of the cartesian referential of all videos. The known dimensions of the A4 were further used to convert coordinates from pixels to millimetres.
- **OpLan_C2_remove_impossibilities.py**: Python code used to account for DeepLabCut tracking errors, with any ant movement exceeding two millimetres per frame being considered implausible and subsequently removed.
- **OpLan_C3_find_changepoints.py**: Python code used to automatically derive the times at which an ant reached and left the reward from the tracking data.
- **OpLan_C4_inward_outward_data.py**: Python code used to calculate relevant measures for the foodward (inward) and nestward (outward) journey such as journey duration, mean instantaneous speed and path tortuosity.
- **OpLan_C5_Figure_2.R**: R code used to produce the raw elements of Figure 2.
- **OpLan_C6_Figure_4.R**: R code used to produce the raw elements of Figure 4.
- **OpLan_C7_Statistical_Analysis.html**: Complete statistical analysis and code for the manuscript.

For the reader's convenience, we highlight the most relevant statistical outputs here. For a comprehensive examination of all statistical analyses, we kindly refer you to the HTML file (OpLan_C7_Statistical_Analysis.html) available in the Zenodo repository.

Foodward Journey Duration

Table S1 – Analysis-of-variance results for the fixed-effects of the foodward journey duration mixed effects cox proportional-hazards model (Related to Figure 2 and 3). Rows highlighted in green reflect "significant" effects based on α = 0.05. Notably, "Treatment" is not "statistically significant". Nevertheless, due to its interaction with "Visit," it must be kept throughout post-hoc analysis.

	Df	Chisq	Pr(>Chisq)
**Treatment**	3	7.5016	0.0575
**Visit**	1	21.7406	0.000003
**Reward_Side**	1	0.0385	0.8445
**Treatment:Visit**	3	17.0190	0.0007

Table S2 – Estimated marginal means of linear trends obtained from the foodward journey duration mixed effects cox proportional-hazards model (Related to Figure 2 and 3). Rows highlighted in green reflect significant effects based on the estimated confidence intervals.

Contrast	Estimate	SE	df	asymp.LCL	asymp.UCL
**Control – Nothing**	5.6	6.5	Inf	-12.6	23.8
**Control – 25ppm**	-22.3	10.2	Inf	-50.9	6.3
**Control – 250ppm**	-38.0	10.8	Inf	-68.2	-7.8
**Control – 2000ppm**	2.4	10.6	Inf	-27.2	32.1
**25ppm – Nothing**	27.8	7.9	Inf	5.7	50.0
**25ppm – 250ppm**	-15.7	11.6	Inf	-48.3	16.8
**25ppm – 2000ppm**	24.7	11.5	Inf	-7.6	57.0
**250ppm – Nothing**	43.5	8.6	Inf	19.5	67.6
**250ppm – 2000ppm**	40.4	12.1	Inf	6.6	74.2
**2000ppm – Nothing**	3.1	8.4	Inf	-20.5	26.8

Nestward Journey Duration

Table S3 – Analysis-of-variance results for the fixed-effects of the nestward journey duration mixed effects cox proportional-hazards model (Related to Figure 4 and 5). Rows highlighted in green reflect “significant” effects based on α = 0.05.

	Df	Chisq	Pr(>Chisq)
**Treatment**	3	1.7819	0.6189
**Visit**	1	6.2049	0.0127
**Reward_Side**	1	0.1548	0.6940
**Treatment:Visit**	3	1.6349	0.6515

Table S4 – Estimated marginal means of linear trends obtained from the nestward journey duration mixed effects cox proportional-hazards model (Related to Figure 4 and 5). Rows highlighted in green reflect significant effects based on the estimated confidence intervals.

	Estimate	SE	df	asymp.LCL	asymp.UCL
**Visit Trend**	11.0	4.2	Inf	2.8	19.2

Foodward Mean Instantaneous Speed

Table S5 – Analysis-of-variance results for the fixed-effects of the foodward mean instantaneous speed linear mixed effects model (Related to Figure 2 and 3). Rows highlighted in green reflect "significant" effects based on $\alpha = 0.05$.

	Df	Chisq	Pr(>Chisq)
**Treatment**	3	1.4096	0.7033
**Visit**	1	8.0195	0.0046
**Reward_Side**	1	0.1608	0.6884
**Treatment:Visit**	3	1.8448	0.6052

Table S6 – Estimated marginal means of linear trends obtained from the foodward mean instantaneous speed linear mixed effects model (Related to Figure 2 and 3). Rows highlighted in green reflect significant effects based on the estimated confidence intervals.

	Estimate	SE	df	lower.CL	upper.CL
**Visit Trend**	0.34	0.12	389	0.10	0.59

33 Nestward Mean Instantaneous Speed

Table S7 – Analysis-of-variance results for the fixed-effects of the nestward log(mean instantaneous speed) linear mixed effects model (Related to Figure 4 and 5). Rows highlighted in green reflect “significant” effects based on α = 0.05. Notably, "Treatment" and “Visit” are not “statistically significant” on their own. Nevertheless, due to their interaction, they must be kept throughout post-hoc analysis.

	Df	Chisq	Pr(>Chisq)
**Treatment**	3	1.6535	0.6473
**Visit**	1	1.5456	0.2138
**Reward_Side**	1	2.0395	0.1533
**Treatment:Visit**	3	10.1965	0.0170

Table S8 – Estimated marginal means of linear trends obtained from the nestward log(mean instantaneous speed) linear mixed effects model (Related to Figure 4 and 5). Rows highlighted in green reflect significant effects based on the estimated confidence intervals.

Contrast	Estimate	SE	df	lower.CL	upper.CL
**Control – Nothing**	0.01	0.02	391	-0.03	0.06
**Control – 25ppm**	-0.01	0.02	389	-0.08	0.06
**Control – 250ppm**	-0.03	0.03	389	-0.10	0.04
**Control – 2000ppm**	0.05	0.02	390	-0.02	0.12
**25ppm – Nothing**	0.02	0.02	388	-0.03	0.07
**25ppm – 250ppm**	-0.03	0.03	388	-0.10	0.05
**25ppm – 2000ppm**	0.06	0.03	388	-0.02	0.14
**250ppm – Nothing**	0.05	0.02	388	-0.01	0.10
**250ppm – 2000ppm**	0.08	0.03	389	0.01	0.16
**2000ppm – Nothing**	-0.04	0.02	389	-0.09	0.02

34

Foodward Path Tortuosity

Table S9 – Analysis-of-variance results for the fixed-effects of the foodward log(path tortuosity) linear mixed effects model (Related to Figure 2 and 3). Rows highlighted in green reflect "significant" effects based on $\alpha = 0.05$. Notably, "Treatment" is not "statistically significant". Nevertheless, due to its interaction with "Visit", it must be kept throughout post-hoc analysis.

	Df	Chisq	Pr(>Chisq)
**Treatment**	3	1.7423	0.6276
**Visit**	1	54.9648	0.0000000
**Reward_Side**	1	4.1340	0.0420
**Treatment:Visit**	3	7.8120	0.0501

Table S10 – Estimated marginal means of linear trends obtained from the foodward log(path tortuosity) linear mixed effects model (Related to Figure 2 and 3). Rows highlighted in green reflect significant effects based on the estimated confidence intervals.

Contrast	Estimate	SE	df	lower.CL	upper.CL
**Control – Nothing**	-0.40	0.14	396	-0.80	0.01
**Control – 25ppm**	0.51	0.22	392	-0.12	1.14
**Control – 250ppm**	0.43	0.23	393	-0.22	1.07
**Control – 2000ppm**	0.02	0.23	395	-0.62	0.67
**25ppm – Nothing**	-0.91	0.17	390	-1.39	-0.42
**25ppm – 250ppm**	-0.08	0.25	390	-0.78	0.62
**25ppm – 2000ppm**	-0.49	0.25	391	-1.18	0.21
**250ppm – Nothing**	-0.83	0.18	390	-1.33	-0.32
**250ppm – 2000ppm**	-0.41	0.25	392	-1.11	0.30
**2000ppm – Nothing**	-0.42	0.18	393	-0.92	0.08

<u>Nestward Path Tortuosity</u>

Table S11 – Analysis-of-variance results for the fixed-effects of the nestward log(path tortuosity) linear mixed effects model (Related to Figure 4 and 5). Rows highlighted in green reflect “significant” effects based on $\alpha = 0.05$.

	Df	Chisq	Pr(>Chisq)
**Treatment**	3	5.3508	0.1478
**Visit**	1	7.5014	0.0062
**Reward_Side**	1	0.1532	0.6955
**Treatment:Visit**	3	1.2631	0.7379

Table S12 – Estimated marginal means of linear trends obtained from the nestward log(path tortuosity) linear mixed effects model (Related to Figure 4 and 5). Rows highlighted in green reflect significant effects based on the estimated confidence intervals.

	Estimate	SE	df	lower.CL	upper.CL
**Visit Trend**	-0.07	0.02	390	-0.11	-0.02
